# Antioxidant Capacity of Melatonin on Preimplantation Development of Fresh and Vitrified Rabbit Embryos: Morphological and Molecular Aspects

**DOI:** 10.1371/journal.pone.0139814

**Published:** 2015-10-06

**Authors:** Gamal M. K. Mehaisen, Ayman M. Saeed, Ahmed Gad, Ahmed O. Abass, Mahmoud Arafa, Ashraf El-Sayed

**Affiliations:** 1 Department of Animal Production, Faculty of Agriculture, Cairo University, Giza, Egypt; 2 Department of Animal Biotechnology, Animal Production Research Institute, Dokki, Giza, Egypt; 3 Cairo University Research Park (CURP), Faculty of Agriculture, Cairo University, Giza, Egypt; 4 Animal Health Research Institute, Dokki, Giza, Egypt; Rutgers University -New Jersey Medical School, UNITED STATES

## Abstract

Embryo cryopreservation remains an important technique to enhance the reconstitution and distribution of animal populations with high genetic merit. One of the major detrimental factors to this technique is the damage caused by oxidative stress. Melatonin is widely known as an antioxidant with multi-faceted ways to counteract the oxidative stress. In this paper, we investigated the role of melatonin in protecting rabbit embryos during preimplantation development from the potential harmful effects of oxidative stress induced by *in vitro* culture or vitrification. Rabbit embryos at morula stages were cultured for 2 hr with 0 or 10^−3^ M melatonin (C or M groups). Embryos of each group were either transferred to fresh culture media (CF and MF groups) or vitrified/devitrified (CV and MV groups), then cultured *in vitro* for 48 hr until the blastocyst stage. The culture media were used to measure the activity of antioxidant enzymes: glutathione-s-transferase (GST) and superoxide dismutase (SOD), as well as the levels of two oxidative substrates: lipid peroxidation (LPO) and nitric oxide (NO). The blastocysts from each group were used to measure the expression of developmental-related genes (GJA1, POU5F1 and Nanog) and oxidative-stress-response-related genes (NFE2L2, SOD1 and GPX1). The data showed that melatonin promoted significantly (P<0.05) the blastocyst rate by 17% and 12% in MF and MV groups compared to their controls (CF and CV groups). The GST and SOD activity significantly increased by the treatment of melatonin in fresh or vitrified embryos, while the levels of LPO and NO decreased (P<0.05). Additionally, melatonin considerably stimulated the relative expression of GJA1, NFE2L2 and SOD1 genes in MF and MV embryos compared to CF group. Furthermore, melatonin significantly ameliorated the reduction of POU5F1 and GPX1 expression induced by vitrification. The results obtained from the current investigation provide new and clear molecular aspects regarding the mechanisms by which melatonin promotes development of both fresh and vitrified rabbit embryos.

## Introduction

Livestock systems occupy about 30% of the planet's ice-free terrestrial surface area [1] and are a significant global asset with a value of at least $1.4 trillion. Several disease outbreaks and climate challenges threaten livestock permanence. Embryo cryopreservation is an essential tool that could be used to reconstitute livestock populations, particularly in endangered species and/or spread valuable genetic resources. Vitrification is an economical method for embryo cryopreservation that permits the rapid cooling without ice crystal formation [2–5]. However, vitrification carries the risk of exposure to oxidative stress derived by excessive free radicals [6–8]. The oxidative stress induced by vitrification could be due to suppression of embryos’ a defensive capacity against reactive oxygen species (ROS) [9]. Normally, the production of oxidant molecules like ROS is counterbalanced by antioxidants such as glutathione and vitamins C and E, as well as by enzymes such as catalase, superoxide dismutase, and glutathione peroxidase that convert ROS to less-damaging molecules [10]. Overproduction of ROS induced by vitrification is detrimental for the embryos due to different types of cell injuries, including membrane lipid peroxidation, impaired intracellular milieu, disturbed metabolism, amino acid and nucleic acid oxidation, adenosine triphosphate (ATP) depletion, mitochondrial dysfunction, apoptosis and necrosis [11–14]. These negative consequences of oxidative stress suppressed gene expression involved in *in vitro* embryo development. Expression of genes responsible for compaction and cell-to-cell adhesion, such as GJA1 (CX43), were found to be decreased when embryos produced *in vitro* compared with *in vivo* [15,16]. Also, low expression of GJA1 in blastocyst was associated with the low quality and survival after cryopreservation [17]. It was evidenced that POU5F1/Oct4 is essential for early development of mouse and human embryos [18,19]. Low expression of OCT3/4 genes in *in vitro*-produced blastocysts is associated with the low pluripotency [20] and/or reduction in ICM cells [21]. It was also reported that POU5F1 and Nanog genes are key regulators in proliferation and differentiation in preimplantation embryos [22]. Nanog gene was also reported to be an important gene for pluripotency and maintaining the stem cell state in rodent embryonic stem cells [23,24].

Melatonin (N-aceyl-5-methoxytryptamine) is produced mainly by the pineal gland and has an important role in controlling ROS [25]. One reason melatonin is such an effective antioxidant is that it does not act through a single mechanism, instead functions in a multifactorial manner to counteract oxidative stress. For example, melatonin acts as a direct scavenger of toxic oxygen derivatives and has the ability to reduce the formation of reactive species [26,27]. It also stimulates the gene expression or activity of other antioxidant enzymes and thus prevents the damage that may occur as a result of oxidative stress [28]. Many researchers confirmed that melatonin enhances *in vitro* development of embryos in mouse [29–32], porcine [33–35], ovine [14,36], bovine [37–39], buffalo [40] and rabbits [41]. The beneficial effects of melatonin on embryonic development were attributed to its ability to down regulate the expression of pro-apoptic genes (BAX and Caspase–3), up regulate the expression of an anti-apoptic gene (Bcl–2), and neutralize the effects of ROS [26,42,43]. In addition, it was found that treatment with melatonin in porcine parthenogenetic blastocysts increased the expression of OCT4 gene [44].

Few studies were carried out concerning protective effects of melatonin on cryopreserved and thawed embryos. For example, Abecia et al. [36] reported that melatonin improved the survival of thawed ovine embryos, increased the rate of hatched embryos and reduced the rate of degenerated embryos at the end of *in vitro* culture. Recently, Succu et al. [14] found a positive effect for 10^−9^ M melatonin on the development of vitrified ovine embryos during post-warming culture. In addition, Dehghani-Mohammadabadi et al. [13] concluded that melatonin at 10^−12^ M increased the cleavage rate, blastulation, and number of ICM and TE cells, and in contrast, decreased the apoptic index in mice vitrified embryos. These data suggest that melatonin may be particularly effective for increasing cryotolerance of vitrified embryos during thawing and *in vitro* manipulation. However, most of these studies focused on morphological aspects of embryo development and gaps remain to understand the extraordinary effect of melatonin at intracellular or molecular levels. Therefore, this study was carried out to investigate the beneficial effects of melatonin on preimplantation development of fresh and vitrified rabbit embryos at morphological and molecular levels.

## Materials and Methods

### Chemicals

Except when mentioned specifically, melatonin and other experimental reagents were purchased from Sigma-Aldrich (S.A., Egypt).

### Animals and ethics statement

A total of 30 nulliparous rabbit does and 10 bucks belonging to the Red Baladi breed [45] were purchased from the rabbit farm stations of Animal Production Research Institute (APRI, Cairo, Egypt). All does and bucks were housed during the study period in a semi-closed rabbitry housing system (Agricultural Experiment Station, Faculty of Agriculture, Cairo University) and kept in batteries of individual cages (60x50x35 cm), supplied with feeding hoppers made of galvanized steel sheets and nipples for automatic drinker. They were maintained under the same standard environmental conditions with light alternating on a cycle of 16 light hours and 8 dark hours, fed with the same commercial diet (18.4% CP, 3.1% ether extract, 12.7% crude fibre and 2.600 kcal DE/kg) and had free access to water. All the experimental protocols were approved by the Research Ethics Committee at the Faculty of Agriculture, Cairo University.

### Embryo recovery

Females were synchronized for the receptivity by an intramuscular injection with 20 IU eCG (Folligon, Intervet, Netherland) 60 hr before insemination. Does were inseminated with semen from adult males of the same breed as described by Lavara et al. [46]. Seventy two hours later, does were sacrificed by intravenous injection of a 1% solution of sodium thiobarbital and embryos were immediately collected by uterine flushing at room temperature (20–25°C). Flushing media consisted of DPBSCa (0.132 g calcium chloride/ 1 liter of Dulbecco’s phosphate-buffered saline), supplemented with 2 g Bovine Serum Albumin (BSA) and 10 ml antibiotics (10,000 units penicillin-G and 10 mg streptomycin per ml, penicillin-streptomycin solution 100X, BioShop Canada Inc.).

### Treatments and embryo culture

Only normal recovered embryos (compact morula with intact mucin coat and zona pellucida) from each female were cultured *in vitro* for 2 hr in a one-well embryo culture dish (NUNC A/S, Thermo Fischer Scientific, Roskilde Site, Denmark), containing 3 ml culture media (TCM199 + 20% fetal bovine serum + 1% antibiotics) supplemented with 10^−3^ M of melatonin (M) or without melatonin supplementation to serve as control (C). Afterwards, embryos of each group were either transferred to fresh culture media and collected as fresh blastocysts (CF and MF groups), or directly vitrified/devitrified according to the methodology previously described by Mehaisen et al. [5] and cultured *in vitro* until blastocyst stage (CV and MV groups). Embryo culture was conducted in a 4-well embryo culture dish (10 embryos per well contained 1 ml of culture media; NUNC A/S, Thermo Fischer Scientific, Roskilde Site, Denmark) at 38.5°C, 5% CO2 and saturated humidity. Following 48 hr in culture media, the developmental ability of each group was calculated as the percentage of embryos that reached either the hatched or expanded blastocyst stages. Thereafter, developed blastocysts from each group were directly collected for evaluating gene expression profile, while the remaining culture media were used for measuring the activity of antioxidant enzymes and the levels of oxidative substrates released by the embryos.

### Antioxidant enzymes activity and oxidative substrates levels

After embryo culture, 4 samples of culture media, 1 ml each, from each group were immediately frozen at -20°C for later analysis. The activity of two antioxidant enzymes: glutathione-s-transferase (GST) and superoxide dismutase (SOD), as well as levels of two oxidative substrates: lipid peroxidation (LPO) and nitric oxide (NO), were analyzed using colorimetric assay kits (K263-100 for GST, K335-100 for SOD, K739-100 for LPO and K262-200 for NO; BioVision, Inc., Milpitas, USA). The standard curves and calculations were performed following the kits protocol for each analysis then all values were related to the number of embryos developed to blastocyst stage in each sample.

The activity of GST enzyme was determined according to methods described by Habig et al. [47]. Briefly, 50 μl of each sample was added to 55 μl reaction mixture contained 49 μl GST assay buffer (pH 6.5), 1 μl 1-chloro–2,4-dinitrobenzene (CDNB) solution and 5 μl glutathione (GSH). The increase in absorbance at 340 nm was recorded for 5 min using automatic scanning spectrophotometer. The SOD enzyme activity was assayed by monitoring the inhibition of photochemical reduction of xanthine oxidase (XO) according Beyer and Fridovich [48] approach. In brief, Samples of 20 μl were mixed with 200 μl of WST–1 working solution and 20 μl of enzyme working solution then incubated at 37°C for 20 min. One unit of SOD activity was defined as the amount of enzyme required to cause 50% inhibition of the reduction of XO as monitored at 450 nm.

The LPO levels were determined by measuring the level of thiobarbituric acid reacting substances (TBARS) according to methods described by Farombi et al. [49] with modification. Briefly, 10 μl of sample was mixed with 500 μl of 10% trichloroacetic acid (TCA) containing 0.01 ml 5% (w/v) butylatedhydroxytoluene (BHT) and 500 μl of 0.75% TBA in 0.1 M HCl. The mixture was heated at 95°C for 60 min and after cooling centrifuged for 10 min at 10,000 *g*. The absorbance of Malondialdehyde (MDA) in the supernatant was determined using an automatic scanning spectrophotometer at 532 nm. The levels of NO were detected by colorimetric analysis in a simple two-step process [50]. In the first step, 85 μl of the sample was mixed with 5 μl of the Nitrate Reductase and 5 μl of the enzyme cofactor then incubated at room temperature for 1 hr to convert nitrate to nitrite. In the second step, 5 μl of the enhancer was added to each sample and incubated for 10 min, followed by 50 μl Griess Reagent R1 (containing phosphoric acid) and 50 μl Griess Reagent R2 (containing NED, N-(1-Naphthyl)ethylenediamine dihydrochloride). The azochromophore color amount developed from the last step was detected using automatic scanning spectrophotometer at 540 nm.

### RNA isolation and quantitative real-time PCR

At the end of embryo culture, 30 blastocysts from each group (three replicates, 10 embryos each) were transferred from culture media into cryogenic vials (Corning Incorporated, Corning, NY, USA) and directly plunged into liquid nitrogen (LN_2_) for later analysis. Total RNA isolation was performed using the Arcturs® PicoPure® RNA isolation kit (Applied Biosystems, Carlsbad, USA) per manufacturer's instruction. Genomic DNA contamination was removed by performing column DNA digestion using RNase-free DNase (Qiagen GmbH, Hilden, Germany). RNA was eluted in 11 μl of elution buffer. The RNA from each replicate was reverse transcribed using 1 mM oligo (dT) primers and the Rever-AidcDNA synthesis kit (Thermo Fisher Scientific, Heidelberg, Germany) per manufacturer’s recommendations. Sequence-specific primers (Table 1) for the real-time PCR were designed using the Primer-blast web interface (http://www.ncbi.nlm.nih.gov/tools/primer-blast/index.cgi) and each pair of primers was tested to achieve efficiencies close to 1.

**Table 1:** Details of primers used for real-time PCR quantitative analysis.

Gene symbol	Gene full name	GenBank accession number	Primer sequences	Annealing temperature (°C)	Product size (bp)
*GJA1*	Gap Junction Protein, Alpha 1	NM_001198948	F:atgagcagtctgcctttcgt	55	228
			R:cgttgacaccatcagtttgg		
*POU5F1*	POU Class 5 Homeobox 1	NM_001099957	F:gagatttgcaaagcggagac	55	188
			R:cggttacagaaccacacacg		
*Nanog*	Nanog Homeobox	XM_002712762	F:gccagtcgtggagtaaccat	55	196
			R:tgtgctgtgttctggctttc		
*NFE2L2*	Nuclear Factor, Erythroid 2-Like 2	XM_002712305	F:tgaaatcctcccaattcagc	55	228
			R:gtgaagactgggctctcgac		
*SOD1*	Superoxide Dismutase 1	NM_001082627	F:cacttcgagcagaagggaac	54	184
			R:cgtgcctctcttcatccttc		
*GPX1*	Glutathione Peroxidase 1	NM_001085444	F:gcttcgagaagttcctggtg	53	218
			R:gcgttcctccatttgttttc		
*GAPDH*	Glyceraldehyde-3-Phosphate Dehydrogenase	NM_001082253	F:aggtcatccacgaccacttc	57	202
			R:gtgagtttcccgttcagctc		
*H2A*	Histone H2A. F/Z variant	NM_001170941	F:cgcttccaaggatctcaaag	56	211
			R:acaatgatggggagaacgag		

qRT-PCR for the 3 replicates of each group has been done in 20 μl reaction volume containing Maxima SYBR Green qPCR Master Mixes with ROX (Thermo Fisher Scientific, Heidelberg, Germany), the cDNA samples and the specific forward and reverse primers in Mx3000P™ real time PCR system (Stratagene). The thermal cycling parameter was set to 95°C for 3 min, 40 cycles at 95°C for 15 s and 60°C for 1 min. After the end of the last cycle, melting curve was generated by starting the fluorescence acquisition at 60°C and taking measurements every 7 s interval until the temperature reached 95°C (S1 Fig). The comparative cycle threshold (CT) method was used to quantify expression levels as described by Bermejo-Alvarez [51].

### Statistical analysis

All statistical analyses were performed using IBM SPSS 22.0 Software Package (IBM corp., NY, USA, 2013). A generalized linear model was performed using a binary probit model with binomial distribution to analyze differences in the *in vitro* development rates in embryo groups (CF, CV, MF and MV). One-way ANOVA was used to determine statistical differences in antioxidant enzymatic activity between embryo groups followed by a multiple pair wise comparison (Duncan’s test). Gene expression data between embryo groups were analyzed using one-way ANOVA, followed by a multiple pairwise comparison using t-tests. Results are expressed as means ± S.E.M. and the significance level was set at P<0.05.

## Results

### Embryo culture and *in vitro* development rates

A total of 197 normal embryos at morula stage were recovered and randomly allocated for the treatment groups. Overall data and *in vitro* developmental rates of fresh and vitrified embryos treated or not treated with melatonin are presented in Table 2. Supplementation of culture media with melatonin for 2 hr significantly promoted the blastocyst rate when compared with fresh control embryos (93% vs. 76% for MF vs. CF, P<0.05). It tended to boost the blastocyst rate in vitrified embryos previously treated with melatonin in comparison with control vitrified embryos (81% blastocyst rate in MV group vs. 69% in CV group, P>0.05). In addition, melatonin enhanced the expanding rate of fresh and vitrified embryos even though improvement was not significant, while vitrification significantly increased the expanding rate in CV and MV groups when compared with CF group (64% and 70% vs. 32%, respectively, P<0.05). On the other hand, the hatchability rate was significantly lower in vitrified embryos compared to fresh embryos (4% and 11% for CV and MV groups vs. 44% for both CF and MF groups, respectively, P<0.05).

**Table 2:** *In vitro* development rates of fresh and vitrified rabbit embryos previously cultured with 0 or 10^−3^ M melatonin.

Embryo groups	N	*Blastocyst rate (Means ± SE)	*Expanding rate (Means ± SE)	*Hatchability rate (Means ± SE)
**CF**	50	0.76±0.060 ^b^	0.32±0.066 ^c^	0.44±0.070 ^a^
**MF**	55	0.93±0.035 ^a^	0.49±0.067 ^b^	0.44±0.067 ^a^
**CV**	45	0.69±0.069 ^b^	0.64±0.071 ^a^ ^b^	0.04±0.031 ^b^
**MV**	47	0.81±0.057 ^a^ ^b^	0.70±0.067 ^a^	0.11±0.045 ^b^

^a, b^ values with different letters in the same column are significantly different (P<*0*.*05*).

N: number of cultured embryos.

* Calculated as a percentage of cultured embryos.

CF: fresh embryos without melatonin, MF: fresh embryos treated with melatonin, CV: vitrified embryos without melatonin, and MV: vitrified embryos treated with melatonin.

### Antioxidant enzymes activity and oxidative substrates levels

Results of antioxidant enzymes activity and the levels of oxidative substrates in culture media of fresh and vitrified embryos supplemented with or without melatonin are shown in Table 3. Melatonin promoted the activation of the antioxidant enzymes in fresh embryos (6.7 vs. 5.1 μM GST/embryo, and 2.1 vs. 0.7 units SOD/embryo in MF vs. CF groups, respectively, P<0.05) and in vitrified embryos (6.3 vs. 4.3 μM GST/embryo, and 1.7 vs. 0.6 units SOD/embryo in MV vs. CV groups, respectively, P<0.05). On the contrary, the levels of oxidative substrates considerably decreased in fresh or vitrified embryo groups treated with melatonin when compared with their controls (LPO: 0.3 vs. 0.7 and 0.5 vs. 1.0 nM/embryo; NO: 0.7 vs. 1.1 and 0.6 vs. 1.4 μM/embryo for MF vs. CF and MV vs. CV groups, respectively, P<0.05).

**Table 3:** Antioxidant enzymes activity and oxidative substrates levels in culture media of fresh and vitrified rabbit embryos previously cultured with 0 or 10^−3^ M melatonin.

Embryo groups	GST μM/embryo	SOD Unit/embryo	LPO nM/embryo	NO μM/embryo
**CF**	5.1±0.50 ^b^	0.7±0.13 ^c^	0.7±0.05 ^b^	1.1±0.06 ^a^
**MF**	6.7±0.38 ^a^	2.1±0.12 ^a^	0.3±0.03 ^d^	0.7±0.07 ^b^
**CV**	4.3±0.28 ^b^	0.6±0.06 ^c^	1.0±0.03 ^a^	1.4±0.15 ^a^
**MV**	6.3±0.22 ^a^	1.7±0.15 ^b^	0.5±0.04 ^c^	0.6±0.09 ^b^

^a, b, c^ values with different letters in the same column are significantly different (P<*0*.*05*).

CF: fresh embryos without melatonin, MF: fresh embryos treated with melatonin, CV: vitrified embryos without melatonin, and MV: vitrified embryos treated with melatonin.

GST: glutathione-s-transferase, SOD: superoxide dismutase, LPO: lipid peroxidation, and NO: nitric oxide.

### Gene expression analysis

**Fig 1:**
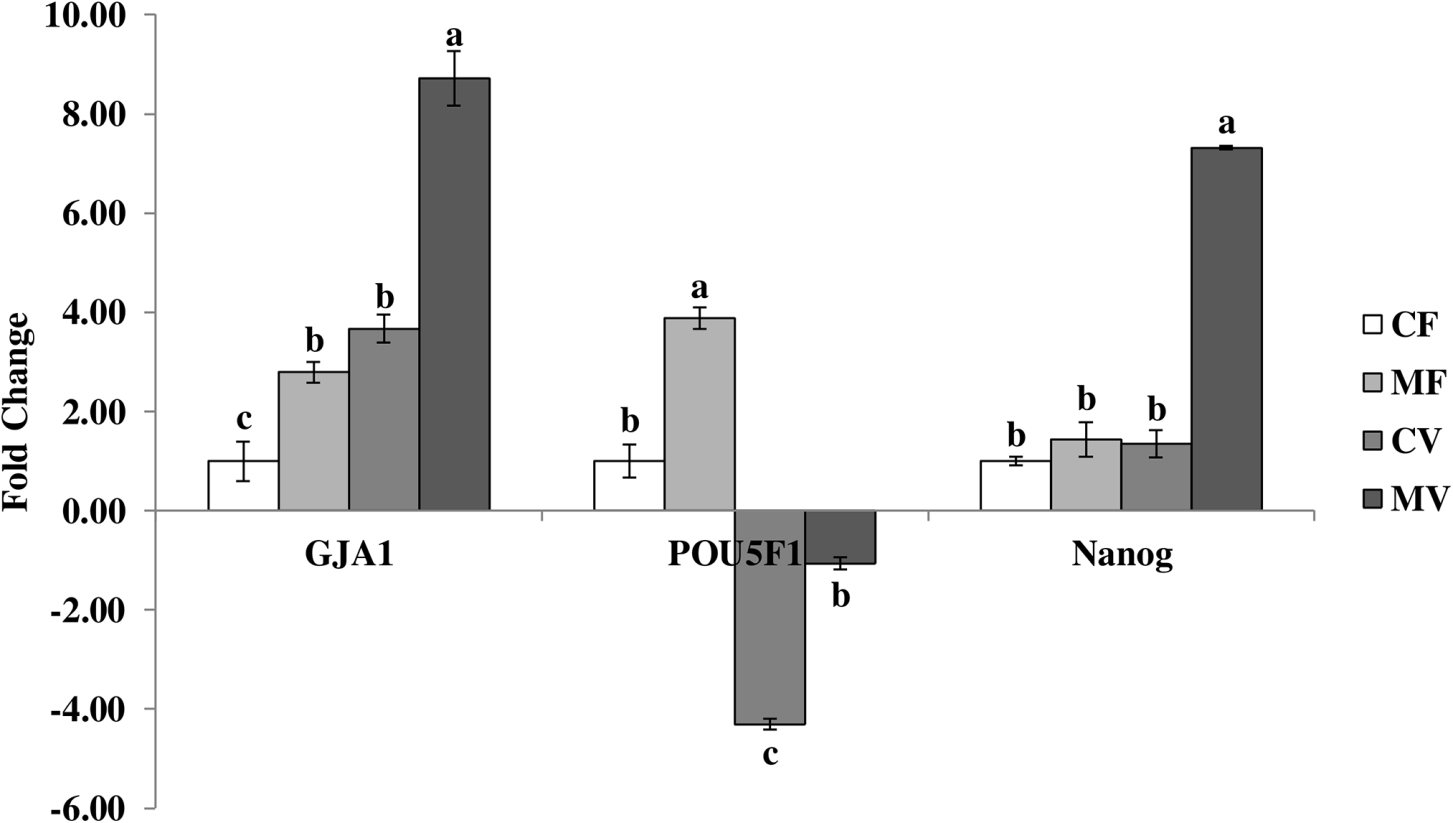
Expression of developmental-related genes in fresh and vitrified rabbit embryos previously cultured with 0 or 10^−3^ M melatonin. CF: fresh embryos without melatonin, MF: fresh embryos treated with melatonin, CV: vitrified embryos without melatonin, and MV: vitrified embryos treated with melatonin; ^a, b, c^ Bars with different superscripts are significantly different (P< 0.05).

Results of quantitative real-time PCR for the examined developmental-related genes GJA1, POU5F1 and Nanog in fresh and vitrified embryos after melatonin supplementation to culture media are illustrated in Fig 1. Vitrification induced significant high expression of GJA1 gene by 3.67 fold (P<0.05) compared to fresh embryos (CF). Furthermore, media supplemented with melatonin resulted in significant stimulation of the GJA1 gene expression in fresh and vitrified embryos (P<0.05). The GJA1 gene was highly expressed in MV (8.72-fold), CV (3.67-fold), and MF (2.79-fold) groups compared to the CF group. The current data shows that the expression of POU5F1 and Nanog genes exibits different patterns. POU5F1 gene expression increased by 3.88 fold in MF embryos group while it decreased by 4.31 and 1.07 fold in CV and MV embryo groups compared to CF group (P<0.05). On the other hand, the expression of Nanog gene was considerably higher in MV group (7.32-fold, P<0.05) than in other embryo groups, while MF or CV groups did not show any significant difference in Nanog gene expression compared to CF embryos group (Fig 1).

**Fig 2:**
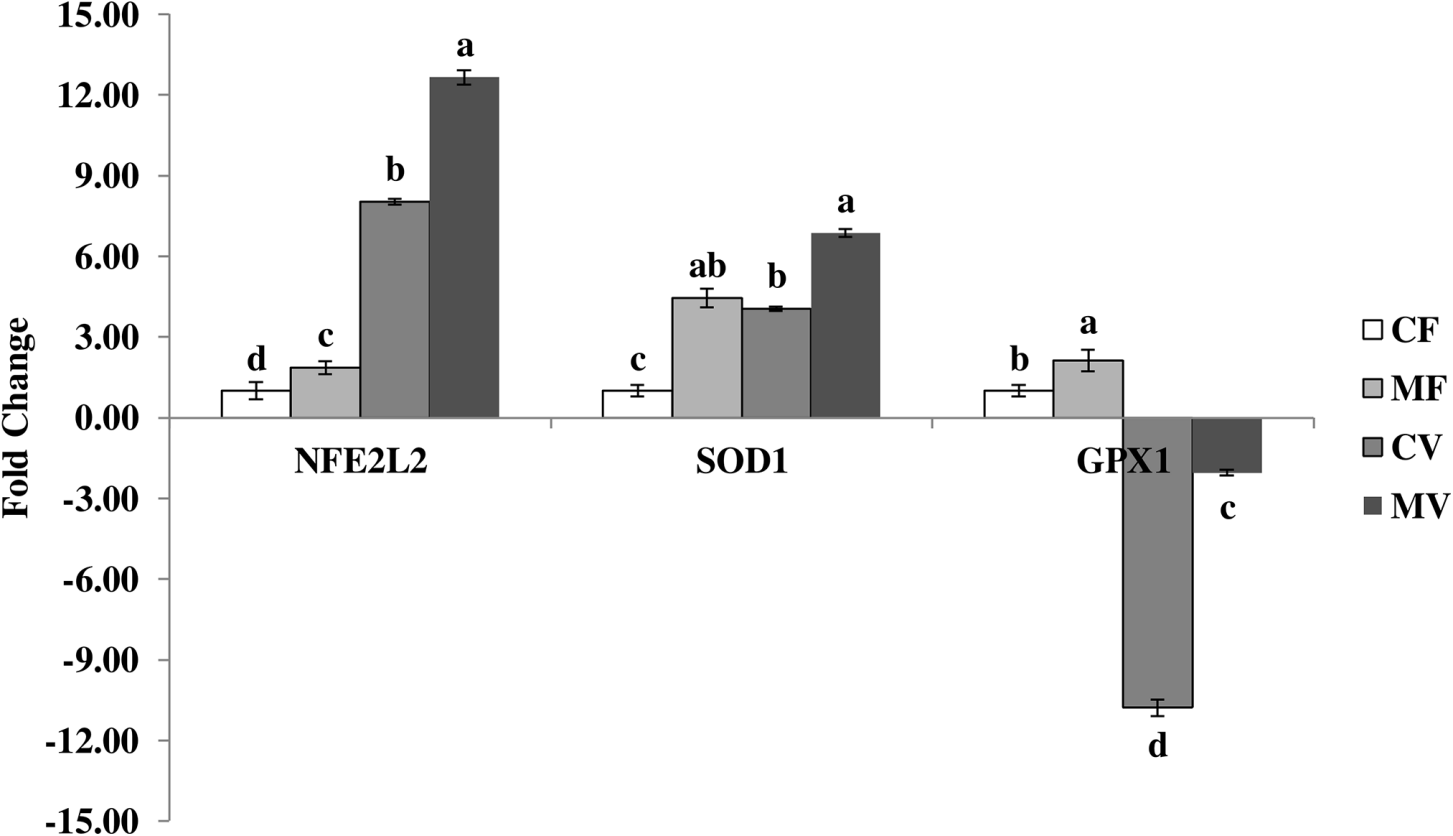
Expression of oxidative-stress-response-related genes in fresh and vitrified rabbit embryos previously cultured with 0 or 10^−3^ M melatonin. CF: fresh embryos without melatonin, MF: fresh embryos treated with melatonin, CV: vitrified embryos without melatonin, and MV: vitrified embryos treated with melatonin; ^a, b, c, d^ Bars with different superscripts are significantly different (P< 0.05).

The expression of oxidative-stress-response-related genes NFE2L2, SOD1 and GPX1 by qRT-PCR in fresh and vitrified embryos after melatonin supplementation to culture media are represented in Fig 2. In general, fresh embryos cultured in media supplemented with melatonin showed a significant high expression of NFE2L2, SOD1 and GPX1 genes in comparison with control embryos (P<0.05). Furthermore, NFE2L2 and SOD1 genes display similar pattern in responding to vitrification or melatonin treatment. A substantial increase in NFE2L2 expression was found in MF, CV and MV groups (1.86, 8.03, and 12.65 fold, respectively) when compared to CF group (P<0.05). Also, the expression of SOD1 gene significantly increased in MF, CV and MV groups by 4.45, 4.05, and 6.87 fold, respectively, compared to CF group (P<0.05). A total of 2.12 fold increase was observed in expression of GPX1 gene in MF embryos in comparison with CF embryos. While a reduction in GPX1 expression was observed in both vitrified embryo groups in comparison with CF group (P<0.05); the decrease was markedly observed in CV and MV groups (10.78 and 2.05 fold, respectively) compared to CF group (Fig 2).

## Discussion

Previous studies mainly discussed the melatonin’s effects on the morphological variations regarding the *in vitro* embryo development, such as cleavage rate, blastocyst rate, hatched blastocyst rate and blastocyst cell number [26,36,52], while few studies were carried out to investigate melatonin effects at cellular and molecular levels in fresh and vitrified embryos, and rabbits were rarely used as animal models. The clear description of embryo development and functional genomic tools in rabbits make them particularly suitable and an important animal model for research for humans and other species [53].

The physiological concentrations of melatonin in plasma of rabbits were previously estimated by radioimmunoassay (RIA) as 22.7 pg/ml [54], that is approximately equivalent to 10^−10^ M. In our previous work [41], we used a concentration of melatonin close to physiological levels (10^−9^ M) and higher concentrations (10^−6^ M and 10^−3^ M) to study its effect on development of rabbit embryos recovered at different stages. It is concluded from this study that a 10^−3^ M concentration of melatonin for morulae embryos recovered from rabbit does at 72 hr post-insemination is optimal. In the present study, supplementation of culture media with 10^−3^ M melatonin also improved the *in vitro* development rate of rabbit embryos (Table 2). We found that blastocyst rate significantly increased by 17% to reach 93% in the MF group compared to 76% in the CF group. The positive effects of the lower doses (10^−7^–10^−12^ M) of melatonin on blastocyst formation and *in vitro* development were also observed in embryos of other species [14,26,32,35,38]. When embryos vitrified, the blastocyst rate decreased to 69% but it tended to increase again to 81% when vitrified embryos were previously treated with melatonin, even though the difference were not statistically significant (Table 2). However, the current study showed that melatonin treatment did not affect the hatchability or expanding of blastocysts like vitrification did itself; hatchability rates ranged between 4–11% vs. 44% and expanding rates ranged between 64–70% vs. 32–49% in vitrified vs. fresh embryos, respectively (P<0.05). The results of the current study are consistent with previous findings which indicated that timing of blastocoel cavity re-expansion after vitrification/warming and *in vitro* culture is associated with the capacity of embryos to restore vitrification injuries, and it is a reliable marker of the development capacity of vitrified embryos [55].

A primary function of melatonin is to serve as a potent-free radical scavenger and a broad-spectrum antioxidant [42,56–58]. The current research extended the points of melatonin investigation on embryo *in vitro* development to include the activity of many antioxidant enzymes. Therefore, LPO and NO levels as oxidative substrates, as well as the activity of GST and SOD as antioxidant enzymes, were evaluated in all experimental groups. In the CV group, low blastocyst rate synchronized with low hatchability was observed after thawing and culture (Table 2). In the same group, the highest level of LPO was recorded (P<0.05), indicating that these embryos were extremely sensitive to damage induced by vitrification and the high formation of LPO and NO released from damaged cells [59]. As shown in Table 3, melatonin significantly increased the activities of GST by 1.6 and 2.0 μM/embryo and SOD by 1.4 and 1.1 unit/embryo in MF and MV groups (P<0.05), respectively, compared to their controls. On the contrary, melatonin significantly diminished LPO by 0.4 and 0.5 nM/embryo and reduced NO by 0.4 and 0.8 μM/embryo in culture media of fresh and vitrified groups (P<0.05) compared to their controls, respectively. It has been known that SOD and GST antioxidant enzymes have an important role in catalyzing the conversion of superoxide to hydrogen peroxide (H_2_O_2_) and catalyzing the reduction of peroxide-containing compounds that may otherwise be toxic to the embryos, resulting in reduction of LPO and NO [60,61]. These observations could explain the damage reduction in embryos cultured with melatonin in MF and MV groups, showing a higher competence of these embryos for development to blastocyst stage than CF and CV embryos (Table 2).

Under conditions of cryopreservation, embryos that be blastulae or expand early, show a higher survival and probability of producing live births and therefore are considered to be more viable than embryos showing delayed blastulation or expanding [20]. When an expression analysis covering genes representative of essential events during development was applied, these embryos differed in expression of developmentally important genes [20,62]. The quantitative real-time PCR for examined developmental-related genes in our experimental groups were also studied and revealed important indications. The relative expression of GJA1 gene (known as Connexin CX43 gene) was significantly up-regulated by treatment of melatonin in fresh (2.79-fold in MF vs. 1-fold in CF) and vitrified (8.72-fold in MV vs. 3.67-fold in CV) embryos (Fig 1). However, we observed that GJA1 gene was over expressed in vitrified embryos, showing the highest expression in MV group when compared with other groups (Fig 1). Referring to differences in morphological aspects between embryo groups (Table 2), the expression of GJA1 seems to be inconsistent with the blastocyst formation and hatchability rates in the current study. These results are in line with Cruz et al. [63] finding, who reported that lower expression of GJA1 gene (CX43) does not correspond to morphological or functional differences of blastocyst types. In addition, Houghton et al. [64] concluded that the absence of Cx43 does not prevent implantation or disrupt prenatal development, and the capacity of preimplantation embryos to develop in the absence of Cx43 could be explained by the presence of any of the additional connexins, such as Cx30, Cx31, Cx40 and Cx45 [65]. These data raise the possibility that gap junctional coupling is not an essential aspect of preimplantation development, despite indication from other study that gap junction assembly is developmentally a regulated event [66]. The high expression of GJA1 gene in the MV group could be explained by the role of gap junctional coupling in amplifying the effects of oxidative stress, especially during vitrification and post-warming *in vitro* culture, and its role in transmission of cell death signals [67]. Alternatively, the supplementation of melatonin itself to the MV group may be another reason for the high incidence of GJA1 gene expression which is necessary in protecting cells from apoptotic cell death [68]. On the other hand, previous reports indicated that POU5F1 (also known as Oct4 gene) has a role in pluripotency and developmental competence of preimplantation embryos [20]. In the present study, a significant reduction in expression of POU5F1 was found in vitrified embryos compared to fresh embryos. However, previous culture of these embryos with melatonin significantly improved the relative expression of POU5F1 by 3.88-fold in fresh embryos and ameliorated the reduction induced by vitrification by 3.24-fold in vitrified embryos when compared to their controls (Fig 1). The over-expression of POU5F1 gene occurred by melatonin in the MF and MV groups was consistently correlated with a high expression in blastocyst rates in the same groups compared to the other groups (Table 2). The beneficial effects of melatonin on cleavage and blastocyst formation rates, and the total cell numbers in blastocysts were previously demonstrated in porcine embryos [44]. They also found a positive correlation of melatonin with expression of pluripotency marker (Oct4 gene). Our results also agree with recent results obtained by Wang et al. [38] who concluded that melatonin promoted blastocyst yield and accelerated *in vitro* bovine embryo development. They also added that melatonin improved the quality of blastocysts that was indexed by an elevated cryotolerance and the up-regulated expressions of developmentally important genes. Nanog is one of the earliest expressed set of genes known to control stemness, self-renewal and development of embryonic cells [69], however, Nanog expression could be detected only in inner-cell mass (ICM) of expanded blastocysts [70]. In the present study, the expression of Nanog gene was significantly higher in the MV group (7.32-fold, P<0.05) than in other embryo groups (Fig 1). The highest expanding rates recorded in vitrified embryos treated with melatonin in our study (Table 2) may explain why Nanog expression was higher in MV embryos when compared to the other groups [70]. While vitrification inflicted selective damage to the inner cell mass of embryos and decreased viability after transfer to recipients [71], the presence of melatonin increased the embryo cryotolerance to vitrification deleterious effects [38] as revealed in our study by enhancing the expression of GJA1 and Nanog genes as well as ameliorating the reduction of POU5F1 gene expression in MV group in comparison with CV group (Fig 1).

The previously reported increase in antioxidant enzyme activity caused by melatonin in our study could be explained by the increased levels of mRNA for the examined oxidative-stress-response-related genes. In fresh embryos, the treatment with melatonin significantly increased the relative expression of NFE2L2 (1.86-fold), SOD1 (4.45-fold) and GPX1 (2.12-fold) when compared to control embryos (Fig 2). Our results are in agreement with that obtained by Wang et al. [39] who reported that SOD1 and GPX4 mRNAs were significantly higher in melatonin-treated bovine embryos than that in controls. Similar results of SOD up-regulation expression by melatonin treatment of murine embryos were also previously reported [32]. According to previous reports [72,73], an inactive form of the NFE2L2 (also known as Nrf2) gene is abundant and sequestered to cytoplasm of blastocysts under low oxidative stress conditions. During vitrification, embryos are exposed to severe oxidative stress and have been more sensitive to oxidative stress [8,74]. Under such high oxidative conditions, an inactive form of Nrf2 is released from the cytoplasm into the nucleus where it can activate its target antioxidant genes by binding to their antioxidant response elements [72,73]. In the present study, embryo vitrification induced a significant increase in expression of NFE2L2 to 8.03-fold and 12.65-fold in CV and MV groups, respectively (Fig 2). The up-regulation increase of NFE2L2 transcript during vitrification was reported to protect embryo from induced oxidative stress through preservation of intracellular redox states and is essential to maintain normal embryonic development [75]. Therefore, the transcript abundance of NFE2L2 with the low hatchability rate in vitrified embryos in the present study may be attributed to the high apoptosis induced in vitrified embryos [76]. However, NFE2L2 profile significantly increased in the presence of melatonin in vitrified embryos, indicating the importance of NFE2L2 to restore embryos to be more tolerant to oxidative stress and more competent for development [73]. In parallel to NFE2L2, the SOD1 gene continued to be highly expressed in vitrified embryos (4.05-fold and 6.87-fold in CV and MV groups, respectively). Similar results were obtained by Jahromi et al. [77] who reported an increase in Mn-SOD expression in vitrified/thawed mouse oocytes compared with the control. The enrichment of nuclear Nrf2 protein is accompanied by more abundant antioxidant gene transcripts including SOD1 in competent blastocysts, and subsequently resulted in reduced ROS accumulation [73]. On the other hand, the relative expression of GPX1 significantly decreased after vitrification to 10.78-fold and 2.05-fold in CV and MV groups, respectively (Fig 2). GPX enzymes are essential for the glutathione redox cycle as a major source of protection against low levels of oxidant stress, whereas other antioxidant enzymes, such as catalase, become more significant in protecting against severe oxidant stress [78]. The high affinity and saturation of H_2_O_2_ that have GPX than other antioxidant enzymes [79,80] may explain the low expression of GPX1 in the CV group and the way it restores to higher levels in the MV and MF groups. We also observed that reduction in GPX1 and POU5F1 expression in vitrified groups (Figs 1 and 2) could be related to the lower hatchability rate in vitrified embryos than fresh embryos (Table 2). This result suggests that although GPX has a pivotal role in cell antioxidant protection during high O_2_ tension [81], its expression may decrease due to the decrease in embryo quality and developmental potential [82].

In conclusion, the current data of the present study show that exogenous melatonin enhances the blastocysts rate in fresh and vitrified embryos with multiple mechanisms of improving the embryonic development. One of the most important mechanisms is to modify the expressions of several embryo-developmentally key genes such as GJA1, POU5F1 and Nanog. Another mechanism could use melatonin to induce changes in the production of antioxidant enzymes and oxidative substrates, or to regulate the expression of oxidative-stress-response-related genes such as NFE2L2, SOD1 and GPX1. Thus, the use of melatonin could be a supportive tool, particularly when embryo development is affected by negative factors.

## Supporting Information

S1 Fig Examples of melting curves output for each selected gene in the quantitative real-time PCR analysis. (TIF)
