# Supplementary figures and images for: Antioxidant Capacity of Melatonin on Preimplantation Development of Fresh and Vitrified Rabbit Embryos: Morphological and Molecular Aspects
Source: PLoS One. 2015 Oct 6;10(10):e0139814. doi: 10.1371/journal.pone.0139814 (PMC4595475; doi:10.1371/journal.pone.0139814)

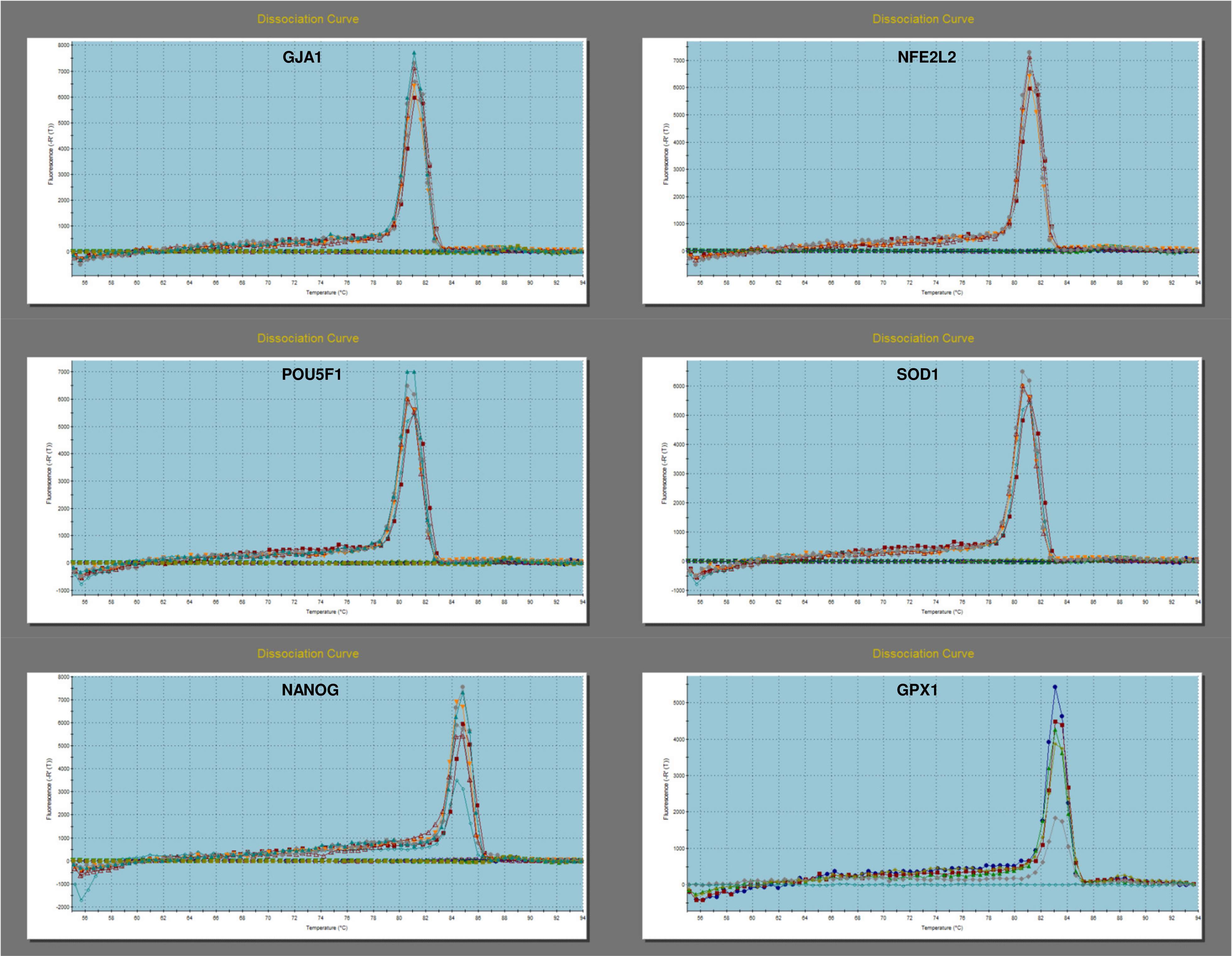

Supplement: S1 Fig — (TIF) [file pone.0139814.s001.tif]
